# Facial aesthetic injections in clinical practice: Pretreatment and posttreatment consensus recommendations to minimise adverse outcomes

**DOI:** 10.1111/ajd.13273

**Published:** 2020-03-22

**Authors:** Greg J. Goodman, Steven Liew, Peter Callan, Sarah Hart

**Affiliations:** ^1^ Monash University Clayton Victoria Australia; ^2^ Shape Clinic Darlinghurst New South Wales Australia; ^3^ Private Practice Geelong Victoria Australia; ^4^ Skin Institute Remuera, Auckland New Zealand

**Keywords:** botulinum toxin, dermal fillers, hyaluronic acid, neuromodulators, rejuvenation

## Abstract

Facial aesthetic treatment with injectable neuromodulators and hyaluronic acid fillers is well established, with favourable safety profiles and consistent outcomes. As with any medical treatment, adverse events and complications may occur. Adverse events associated with these products are typically transient and mild to moderate in severity. Serious adverse events, such as infection and intravascular occlusion, are rare. Proper patient selection, consent and counselling, preparation and impeccable injection technique are important risk reduction strategies. Both clinicians and patients must be alert to the signs and symptoms of complications so that appropriate treatment can be started promptly. In this article, the authors review the current literature and provide their consensus recommendations for minimising adverse outcomes when treating patients with botulinum toxin or hyaluronic acid fillers.

## Introduction

The number of minimally invasive facial aesthetic procedures performed annually continues to increase worldwide.^1^, ^2^ In 2017, more than 8.5 million nonsurgical injection procedures were performed globally, an increase of almost 850 000 from 2015.^3^ Botulinum toxin type A (BoNTA) and hyaluronic acid filler injections are the first and second most common, respectively.^2^, ^3^ Clinical experience with these agents is extensive, and their safety profiles are well characterised.^4^, ^5^, ^6^ Adverse events (AEs) with BoNTA products are generally transient and mild to moderate in severity,^4^, ^5^, ^7^, ^8^, ^9^, ^10^, ^11^ and hyaluronic acid fillers are considered to provide safe, effective and reproducible outcomes.^12^, ^13^ Nevertheless, as the number of complications can be expected to increase with the rising number of procedures performed, clinicians should familiarise themselves with the types of AEs and complications associated with BoNTA products and hyaluronic acid fillers.

The type and frequency of AEs have been well documented in clinical trial publications, product prescribing information and published reviews. In general, overall rates of AEs are similar between BoNTA products, with specific AEs depending, to a certain extent, on the facial treatment area.^4^, ^5^, ^7^, ^8^, ^14^ In our clinical experience, which is consistent with the literature, the most common AEs associated with BoNTA products are related to injection site reactions, such as mild pain, bruising, tenderness and headache.^4^, ^5^, ^9^, ^10^, ^11^, ^15^, ^16^, ^17^

Undesirable effects in patients treated with hyaluronic acid fillers include immediate reactions, such as oedema, and erythema, paraesthesia, pain, bruising and haematoma.^12^, ^18^ Placement‐related undesirable effects include lumps and the Tyndall effect at the injection site. Rarely, more serious complications have been described, such as delayed‐onset nodules, vascular occlusion with resulting tissue necrosis, intravascular blindness and stroke.^12^, ^18^ These can be minimised through knowledge of anatomy, injection training and proper technique, and through careful patient selection, counselling and preparation.

We provide here our consensus recommendations to minimise adverse outcomes with BoNTA products and with hyaluronic acid fillers. It is important to note that, in current medical aesthetic practice, it is increasingly common to treat patients in combination with neuromodulators and fillers.^19^ When administering both modalities in the same session, placement of filler should precede toxin, and the more comprehensive pre‐ and postcare recommendations for fillers should be followed. Principles that apply generally to BoNTA products and to hyaluronic acid fillers are summarised first, followed by product‐specific recommendations.

## General Principles for Facial Injections

A comprehensive understanding of facial anatomy is fundamental to patient safety. Details will not be discussed here, and we refer the reader to several excellent publications for this information.^20^, ^21^, ^22^, ^23^, ^24^ General principles for facial aesthetic injections, including considerations for pretreatment, day of treatment and posttreatment, are shown in Table S1. Of note, it is essential during the pretreatment phase to select patients carefully, manage their expectations for aesthetic outcomes and ensure that they are aware of potential complications.

Before beginning any treatment, it is advisable to prepare a tray with the syringe with the expected dosing and any additional necessary items, such as cleansing agents and antiseptics. Avoiding contamination is essential while preparing patients for treatment and during procedures. A headband may be used to secure the patient’s hair. Two general steps for facial preparation are cleansing and antisepsis. Dirt and make‐up may be removed from the area using a wipe, cleanser or saline. Alcohol wipes or antiseptic‐dampened gauze pads may be used to apply antiseptic solutions. Cotton balls, commonly used in dressing pack, should be avoided as they can leave strands of cotton on the skin.

When using alcohol or chlorhexidine as an antiseptic, caution should be exercised, for they are both extremely irritating to the cornea. Chlorhexidine in particular, when spilled into the eyes, can cause significant chemical burns to the cornea in the form of keratitis, as illustrated by 11 sentinel cases reported in the 1980s.^25^ To minimise risks when using chlorhexidine or alcohol, it is important to prevent excess fluid dripping on the conjunctiva or cornea by using dampened, not soaked, gauze or wipes. A safe and effective alternative to chlorhexidine is povidone–iodine. Another proposed option may be a neutral superoxidised agent, such as a product containing hypochlorous acid, which is not deactivated by bacteria.^26^

Regardless of the choice of antiseptic, it is important to be familiar with and adhere to the Aseptic Non‐Touch Technique (ANTT^®^), sometimes called the no‐touch technique (Figure S1).^27^, ^28^ Considered the ‘de facto international standard for aseptic technique’,^27^ ANTT in the context of facial injections means that the sterilised area should not be touched again, except with the needle. In daily practice, wiping of the treatment area with antiseptic is only advocated if the site where the needle is to be introduced has been touched in the process of stabilising for injection in the adjacent area. This is to avoid contamination from repeated touching of the treatment area during the procedure.

Ideally, surface‐active dermatology procedures, such as laser treatment, should be completed before commencing BoNTA or filler treatments. Otherwise, we recommend that surface‐active procedures be deferred for at most 2 to 4 weeks after filler treatments and 1 day after BoNTA treatment.

## Botulinum Toxin Type A Treatment

### Pretreatment considerations

We strongly recommend that BoNTA treatment be avoided during pregnancy and breastfeeding owing to the lack of adequate data on the developmental risk to a human foetus from the use of BoNTA in pregnant women and evidence of reproductive toxicity in animal studies.^7^, ^8^, ^14^ It is also unknown whether botulinum toxin is excreted in human breastmilk.

The general, multisystem medical review preceding treatment with botulinum toxins should document medication usage, allergies, other planned procedures, and previous use of neuromodulator or filler treatments. The evaluation should ascertain whether the patient has conditions for which there are contraindications, warnings or precautions to botulinum toxins, such as known hypersensitivity reactions to botulinum toxin or to any ingredient in the formulation and the presence of infection at the injection site.^7^, ^8^, ^14^, ^29^ In Australia and New Zealand, BoNTA is contraindicated in patients with myasthenia gravis or other neuromuscular disorders.^30^ To reduce the risk of bruising and ecchymosis, it may be helpful for patients to avoid nonessential over‐the‐counter medications or supplements (e.g. fish oil and vitamin E oil) that may affect blood clotting for approximately 1 week before treatment.^10^, ^31^, ^32^ We suggest that one week is sufficient for stopping nonessential aspirin and other nonsteroidal anti‐inflammatory drug use.

Patient counselling is crucial in several respects, not the least of which is to ensure that patients understand the treatment process and all potential complications sufficiently for them to provide informed consent. Given the popularity and overall general safety of facial aesthetic injections, patients may need to be reminded that these are medical procedures and must be considered seriously. In this regard, patients should be counselled on what to report during and following treatment. In our clinical experience, mild pain, tenderness and stinging are the most commonly reported AEs with BoNTA treatment, but patients should notify clinicians immediately of any posttreatment event that is bothersome or does not resolve. These may include uncommon adverse reactions, such as eyelid or eyebrow ptosis, and inadvertent alteration of the smile by paresis of muscles, such as the zygomaticus major or minor, depressor labii or risorius. Some of these complications can be corrected with injection of BoNTA in muscles that antagonise the affected muscles; in the case of eyelid ptosis, ophthalmic solutions with alpha‐adrenergic effects, such as naphazoline 0.025%/pheniramine 0.3% or apraclonidine 0.5%, may be used to elevate the upper eyelid via contraction of the Müller muscle.^33^

### The treatment process

Pain management is an integral part of facial aesthetic practice, and individuals vary in their sensitivity to pain and in their attitudes and expectations.^34^ Anxiety, the expectation of pain or actual pain may substantially affect patients’ overall experience and may impact their willingness to return for additional treatments. Several pain reduction methods are available. To minimise the onset of pain, injectors should use a slow injection technique with a gentle and slow extrusion rate. Some clinicians use ice or cooling tools before BoNTA injections to reduce pain. Vibration anaesthesia has been shown to be effective in reducing pain for both neuromodulator and filler injections.^35^, ^36^ Others find distraction therapy, such as guided breathing, music or engaging conversation, to be helpful. Icing may also help prevent or minimise posttreatment bruising because it constricts blood vessels. If ice is used, we recommend placing it inside either a sterile glove or a nonsterile glove, then wiping the glove with alcohol or chlorhexidine to sterilise it, as ice is not sterile. Alternatively, frozen gel packs may be used in the same way.

Infection following injections of BoNTA for cosmetic indications is rare and has not been reported in the literature as a treatment‐related event.^4^, ^12^, ^15^, ^18^, ^37^ The authors concur that it is more important to cleanse the skin of dirt and make‐up than to use a disinfectant. Nevertheless, careful preparatory technique can minimise any small risk. Antisepsis of the area to be injected can be undertaken using the antiseptic options and precautions previously described. The skin should be allowed to dry before injections are administered.

Some clinicians choose to mark injection sites, which is traditionally done with a white marker. This optional technique can help keep track of the sequence of injections, particularly if the process is interrupted. Marking should be done before antisepsis, and injections should never be done directly through the white marks. Injecting through a beard or eyebrows is acceptable if those areas have been cleansed thoroughly. Once the injection sites are prepared, ANTT should be used, touching the site only with the needle. Botulinum toxins are supplied and intended as single‐use vials.^7^, ^8^, ^14^ It is critical to avoid interpatient transfer, as highlighted by a report describing the contraction of HIV by 4 patients who received local anaesthetic from a shared vial.^38^ One method utilised by the majority of the authors to minimise cross‐contamination is to extract the entire vial into multiple syringes directly following dilution.

### Posttreatment recommendations

Patients should receive posttreatment instructions in writing, including information on how to contact the office with any after‐hours concerns. We recommend offering a follow‐up appointment 2 weeks posttreatment for patients receiving BoNTA treatments either for the first time or in a new treatment area.

In the authors’ experience, patients may resume normal activities after 4 hours. There is no need to move, or avoid moving, the affected muscles. Some practitioners recommend contracting the treated muscles to facilitate uptake of the product, but this is not necessary. When fillers are used in combination with BoNTA, patients should be advised to follow posttreatment instructions appropriate to their filler treatment (see below).

Recommendations for facial treatments with BoNTA are summarised in Table 1 7, 8, 14, 29 and Table 2.^27^, ^28^

**Table 1:** Pretreatment evaluation and counselling: botulinum toxin type A

Parameter	Steps
Evaluation	Determine whether patient is pregnant or breastfeeding and defer treatment Conduct general multisystem medical history Medications Allergies Recent or planned medical procedures Previous treatment with neuromodulators or filler agents Review contraindications for use,^7^, ^8^, ^14^, ^29^ including the following: Previous hypersensitivity reactions to botulinum toxin or any ingredients in the formulation Infection at the proposed site of injection Myasthenia gravis or other neuromuscular disorders
Counselling	Provide the patient with information regarding what to expect during and after treatments (e.g. mild pain, tenderness or stinging)

**Table 2:** Botulinum toxin type A: recommendations for treatment day and during follow‐up

Parameter	Steps
Treatment day	Conduct a final patient history for contraindications and precautions Prepare the skin for treatment Secure hair, if needed Thoroughly remove dirt and make-up in the cosmetic unit, using mechanical cleansing and/or make-up remover Mark the injection sites with a white marker (optional) Ensure that treatment area is thoroughly cleaned Use gauze rather than cotton balls, as cotton balls may deposit material on the skin; sterile gauze is preferable Use the ANTT^27^, ^28^ Inject BoNTA
Follow‐up	Provide patients with complete posttreatment instructions Be sure patients are aware of any complications that could arise and provide after‐hour contact information in case of questions or concerns

ANTT, Antiseptic Non‐Touch Technique; BoNTA, botulinum toxin type A.

## Hyaluronic Acid Filler Treatments

### Pretreatment considerations

Prospective patients should be carefully evaluated for any existing conditions or medical history that could increase the risk of infection. Risk factors include a history of complications with soft‐tissue fillers or of multiple prior filler treatments, poor periodontal hygiene, existence of an immunocompromised state, chronic or recurrent skin conditions, current herpes labialis or uncontrolled type 2 diabetes mellitus.^39^ We caution that prospective patients’ disclosures may be unreliable with regard to their full medical history, including prior filler procedures, which could skew the diagnosis and proper treatment of infectious reactions. Stable autoimmune conditions are not considered an absolute contraindication to treatment, and patients with normal wound healing may be treated. Individuals with a history of autoimmune disease should be evaluated on a case‐by‐case basis.^32^ We advise delaying treatment for patients who are unwell, for example who have a fever, cold or influenza.

As with all aesthetic procedures, setting expectations for outcomes and reviewing potential AEs is integral to patient counselling. It is essential that all material risks be included in informed consent forms. Patients should be instructed to immediately report the development of nodules, both immediate and delayed, as they may result from varied aetiologies, including improper filler placement, infection or reaction to filler material.^12^ Although vascular occlusion leading to blindness is rare, patients should be explicitly advised about the risks, including all visual complications.^40^, ^41^, ^42^, ^43^, ^44^ Patients receiving injections with hyaluronic acid fillers should be clearly and explicitly educated about the types of visual disturbances that could herald a serious AE, including ocular pain, double vision or blindness in one or both eyes. As vascular occlusion may present up to 72 hours after treatment, it is prudent to forgo treatment of any patient who plans to travel during this time frame under circumstances that could prevent rapid access to hyaluronidase. Patients will need to provide consent to reversal of hyaluronic acid fillers with injections of hyaluronidase.

Patients using prescription anticoagulant and anti‐inflammatory drugs may be at increased risk of bruising. Filler injections may still be used safely in such patients, as the risk:benefit ratio does not favour cessation of these prescription medications. Patients should, however, be advised to stop taking nonessential or elective medications that have anticoagulant effects about a week before treatment.^32^, ^45^, ^46^ Cold compresses and topical vitamin K gel may help ameliorate posttreatment bruising.^45^, ^46^, ^47^, ^48^

#### Contraindications and warnings

The various types of hyaluronic acid fillers have different intended uses, indications and precautions,^49^, ^50^, ^51^, ^52^, ^53^, ^54^, ^55^ and clinicians should review these carefully. None of these products should be used in women who are pregnant or breastfeeding; who have a known hypersensitivity to hyaluronic acid or to gram‐positive bacterial proteins, as hyaluronic acid is produced by *Streptococcus‐*type bacteria; who have a known hypersensitivity to lidocaine or to amide‐type local anaesthetics; who have porphyria; or who are younger than 18 years. These products should not be used in areas presenting with cutaneous inflammatory or infectious processes, such as acne or herpes. These fillers also should not be used simultaneously with laser treatment, deep chemical peels or dermabrasion. For surface peels, it is recommended to postpone injecting the product if the inflammatory reaction generated is substantial. Note that the use of topical tretinoin or oral retinoids is not contraindicated.

### The treatment process

Initial preparation of patients for filler injections is similar to that for BoNTA treatment, including securing hair away from the face and cleansing the face of all dirt and make‐up. If ice or cooling tools are used to reduce pain, they may be used at this time, as previously discussed for BoNTA treatment. The skin may be marked, if desired, and thorough antisepsis should be undertaken, but do not inject filler through the marks. Filler injections should be made slowly, with minimal injection pressure and continuous movement of the needle in the same plane, to minimise inadvertent intravascular placement. Needles should be changed frequently so that they are sharp. To reduce discomfort, topical anaesthesia may be used, particularly for treatments that require a number of skin punctures, such as fine lines, or when treating the lips. When using a local anaesthetic injection as a blockade for the perioral area and lips, anaesthesia is better given as micro‐blebs in the submucosa of the lip sulcus rather than classical infraorbital and mental nerve blocks.

Infection rates associated with soft‐tissue fillers, including hyaluronic acids, are generally low, but the potential for infection may be underappreciated, especially as the number of patients being treated increases.^39^, ^56^, ^57^ Rates appear to vary by the type of filler, patient history, skin preparation methods and injection technique, and range from 0.04% to 0.2%.^39^ A search of the literature failed to reveal clear documentation of infection rates for various types of fillers. An ongoing issue is that these rates typically reflect delayed reactions (inflammatory nodules), the cause of which is not definitively known.^12^, ^47^, ^58^, ^59^ Biofilms have, however, been increasingly implicated as causative.^12^, ^60^ In a mouse model, the sustainability of bacterial infection depended on the longevity of fillers and appeared to result from biofilm formation within the gels.^61^ Immunogenic responses, in addition to biofilms, are also hypothesised to play a role in delayed reactions.^59^

To minimise the risk of contamination and infection, stringent hygiene, including the use of the ANTT at all times, is essential when fillers are injected. Sterile dressing packs or sterile gauze should preferably be used, and it is essential that the gauze is changed frequently because of the potential for ongoing bacterial contamination from the patient’s skin. Note that syringe surfaces are not sterile, underscoring the importance of the ANTT. Because filler injections necessitate using hands to stabilise or pinch the skin, clinicians should perform antisepsis frequently during the procedure. We also note that sterile gloves may provide a false sense of security; therefore, we advocate changing gloves after preparing syringes for injection and marking the face.

Other serious complications, although rare, may result from the inadvertent intra‐arterial injection of any type of hyaluronic acid filler.^42^, ^47^, ^62^, ^63^ Intra‐arterial occlusion may cause tissue necrosis, blindness or stroke following injection,^12^, ^23^, ^42^, ^47^ and intravenous injection may cause nonthrombotic pulmonary embolism.^64^ It is imperative to be alert to the signs and symptoms of vascular complications to enable treatment as soon as possible. Figure S2 provides an overview of some of the signs and symptoms associated with HA filler–induced vision impairment. For example, skin blanching (livedo reticularis) is an immediate or early sign of arterial occlusion and treatment with hyaluronidase should be initiated when hyaluronic acid filler has been used. Critical steps for managing arterial occlusion, including visual complications, were published recently and should be reviewed carefully.^12^, ^41^, ^63^, ^65^, ^66^, ^67^ An emergency flow diagram for recognising retinal occlusion is provided in Figure S3; upon onset of ocular pain and/or vision loss, it is imperative that filler treatment be discontinued immediately and that the patient be prepared for transfer to a hospital setting.^43^, ^68^

Risk reduction strategies and prompt recognition and immediate treatment are essentials in clinical practice.^23^, ^62^ As with all facial aesthetic treatments, in‐depth anatomical knowledge is foundational. Another recommended preventive strategy is injection technique.^18^, ^41^, ^42^, ^48^, ^65^, ^69^ Because of the rapidity with which serious visual impairment can occur, it is essential to restore retinal circulation within 60 to 90 min.^23^, ^42^ Aesthetic practices therefore must ensure that hyaluronidase is readily available to manage complications, such as intravascular injections of hyaluronic acid filler.^12^, ^23^, ^41^, ^42^, ^62^, ^65^ A kit containing the hyaluronidase plus clear instructions for its use and contact information for specialists is invaluable.^65^, ^66^ Although hyaluronidase injection techniques have been described in several published reviews, it is imperative that all injectors be trained on their safe and effective use.

Recommendations for the minimisation of visual intravascular adverse events build upon the overall strategies to minimise the risk of adverse reactions published by Signorini et al.^13^ and include a strong understanding of injection anatomy (including the safest depth for an injection in any given area), then injecting very slowly, with low extrusion pressure. Micro‐boluses should be injected in small aliquots (<0.1 mL) while directing the needle or cannula perpendicular to the primary axial vessels in the anatomical region. To reduce the likelihood of vessel cannulation, move the needle in the chosen plane at all times when delivering micro‐boluses (even if only in small‐amplitude movements), and ensure that the direction of injection is away from the eye in higher‐risk areas, such as the nose, glabella or nasolabial folds.

Cannulas are considered by many to be a safer alternative to a needle in certain areas, including the brow, lateral and anterior cheek, but not for nasal injections. Smaller‐gauge cannulas (<25 #) may behave somewhat like needles in terms of their ability to pierce blood vessels.^70^ Using a local anaesthetic with adrenaline at cannula entry points and in the field to be injected may help to constrict local vessels; however, the patient should be monitored after the injection to ensure that the vasoconstrictive effect resolves, to avoid confusion with intravascular injection of filler. Finally, the use of aspiration before injection is a commonly recommended practice^13^; however, it will not always demonstrate when in a vessel; thus, all other precautions should be utilised by injectors using aspiration.

### Posttreatment recommendations, instructions and follow‐up for patients

Clinicians may massage injection areas gently following treatment if necessary to correct lumps or contours and to ensure the even distribution of the filler.^49^, ^50^, ^51^, ^52^, ^53^, ^54^, ^55^ Some clinicians recommend icing after injection, although this may mask signs of occlusion. Therefore, we do not recommend postinjection icing.

It is essential that injectors, patients and staff be educated about and alerted to symptoms of vascular occlusion, including visual complications, as well as other potential adverse reactions. Posttreatment instructions should reinforce the counselling and education that the patients should have received before treatment. Ideally, recommendations on posttreatment care should be provided in writing and include contact information for after‐hour concerns. Patients experiencing any visual disturbances or increasing pain after treatment should report these events immediately. It is important that whomever the patient calls is aware of the complications and knows how to direct the call, as reassurance over the telephone by anyone is hazardous and needs to be done with absolute knowledge of the situation. All staff, including reception staff, must be educated about this possibility. It is normal to experience some tenderness in the injection area, but increasing pain should be evaluated expeditiously. Other concerns include unusual or worsening bruise‐like appearance within 24 hours of injection or any lesion that presents as a pustule or blister or groups of these within the first 3 postinjection days.

It is normal for patients to experience the sensation of some lumps or firmness for up to 4 weeks. They can be counselled to avoid massaging or rubbing the face or sleeping face‐down for 1 week. Although patients may massage the site after 2 weeks, it is preferable for the injector to address any irregularities on follow‐up. It is also advisable that patients forgo strenuous activity for 24 hours posttreatment. The authors advise that make‐up be used sparingly or, preferably, not at all for the remainder of the day. To reduce the risk of contamination, it is preferable initially for patients to use make‐up from dispensers rather than brushing on products. New patients or patients receiving fillers at new treatment sites should return about 2 to 4 weeks after treatment to evaluate the aesthetic outcomes, at which time touch‐up treatments may be performed.

Recommendations for facial treatments with injectable hyaluronic acid fillers are summarised in Tables 3, 4, 5.^27^, ^28^, ^49^, ^50^, ^51^, ^52^, ^53^, ^54^, ^55^

**Table 3:** Hyaluronic acid tissue filler products: pretreatment evaluation and counselling

Parameter	Steps
Evaluation	Determine whether patient is pregnant or breastfeeding and, if so, defer treatment Conduct general multisystem medical history: Medications Potential risks of infection (e.g. immunocompromised patients, patients with recurrent skin conditions, certain metabolic conditions or autoimmune disease) History of complications with filler treatments History of multiple treatments Review contraindications and warnings/precautions for use,^49^, ^50^, ^51^, ^52^, ^53^, ^54^, ^55^ which include but are not limited to: Pregnancy and lactation Known hypersensitivity to hyaluronic acid and/or gram‐positive bacterial proteins Known hypersensitivity to lidocaine or amide‐type local anaesthetics Presence of active inflammatory or infectious processes, such as acne or herpes

Counselling	Provide the patient with information on what to expect during and after treatments, particularly risks of vascular complications including blindness, as well as mild and transient side effects, such as injection site reactions or posttreatment bruising Advise patients to avoid nonprescribed anticoagulant agents for about 1 week before treatment

**Table 4:** Hyaluronic acid filler products: treatment‐day recommendations

Parameter	Steps
Before beginning treatment	Ensure that hyaluronidase is readily available, that injectors are fully trained to recognise the symptoms of vascular occlusion and that they know how to use hyaluronidase Conduct a final patient history for contraindications and precautions, including establishing whether the patient has an acute infection, such as an upper respiratory tract infection; if so, deter treatment until resolved
Preparing the skin	Secure the patient’s hair, if needed Thoroughly remove dirt and make-up in the cosmetic unit, using mechanical cleansing and/or make-up remover Mark the injection sites with a white marker Apply antiseptic, preferably chlorhexidine, using caution not to splash it into the eyes; pre‐prepared alcohol wipes may also be used Use gauze, preferably sterile, rather than cotton balls, which may deposit material on the skin Allow antiseptics to dry on the skin before injections begin
Injecting	Use the ANTT^27^, ^28^ Inject slowly with minimum injection pressure, utilising constant micro‐movements in the same plane, to minimise intravascular injection Gently massage after injection to smooth any lumps and to ensure even distribution of filler^49^, ^50^, ^51^, ^52^, ^53^, ^54^, ^55^

ANTT, Antiseptic Non‐Touch Technique.

**Table 5:** Hyaluronic acid filler products: patient follow‐up instructions and care

Parameter	Steps
Instructions about complications	Ensure that patients are aware of any complications that may arise Be specific about potentially serious complications, including the following: Increasing pain the day after injection Unusual bruising within 24 hours of injection Pustules or blisters within 3 days of injection Provide after‐hour contact information in case of questions or concerns and ensure that all staff are aware, in case they take the call
General advice (non‐evidence‐based)	Advise patients to avoid: Using make-up for 2 hours after injections Strenuous activity for the first 24 hours Sleeping face-down or rubbing the face for 1 week after treatment Massaging the area for 2 weeks posttreatment; massaging to deal with irregularities should be performed at follow‐up by the injector
Follow‐up	Schedule follow‐up appointments as needed For new patients or for patients treated in a new area, schedule follow‐up 2–4 weeks after treatment

## Conclusions

Injectable BoNTA and hyaluronic acid fillers have well‐established safety profiles. The majority of adverse reactions are transient, self‐resolving and mild to moderate in severity. Many of these events are injection‐related rather than product‐related. Nevertheless, with the continually increasing popularity of minimally invasive facial treatments with neuromodulators and soft‐tissue fillers, clinicians are likely to encounter a greater frequency of rare but serious complications. It is therefore important for clinicians to be fully cognisant of all potential complications, to employ known prevention strategies and to be able to undertake appropriate remedial treatment. Thorough knowledge of anatomy and careful injection techniques are fundamental to achieving optimal outcomes. Owing to the widespread popularity and overall favourable safety profiles of neuromodulator and filler treatments, patients may not appreciate that sometimes serious complications can arise. Comprehensive patient counselling and education are also integral steps in aesthetic practice.

## Supporting information


**Table S1.** General principles on facial injections with botulinum toxin type A and hyaluronic acid filler products.
**Figure S1.** Aseptic Non Touch Technique (ANTT).
**Figure S2.** Recognising the signs and symptoms of vision impairment caused by hyaluronic acid filler injection.
**Figure S3.** Emergency flow diagram for recognising retinal occlusion.
