# Supplementary material for: Facial aesthetic injections in clinical practice: Pretreatment and posttreatment consensus recommendations to minimise adverse outcomes
Source: Australas J Dermatol. 2020 Mar 22;61(3):217–25. doi: 10.1111/ajd.13273 (PMC7497045; doi:10.1111/ajd.13273)
Supplement: Supplementary file 1 — Table S1. General principles on facial injections with botulinum toxin type A and hyaluronic acid filler products. Figure S1. Aseptic Non Touch Technique (ANTT). Figure S2. Recognising the signs and symptoms of vision impairment caused by hyaluronic acid filler injection. Figure S3. Emergency flow diagram for recognising retinal occlusion. [file AJD-61-217-s001.docx]

**Supporting Information**

Additional Supporting Information may be found online in Supporting Information:

**Table S1.** General principles on facial injections with botulinum toxin type A and hyaluronic acid filler products

**Figure S1.** Aseptic Non Touch Technique (ANTT)

**Figure S2**. Recognising the signs and symptoms of vision impairment caused by hyaluronic acid filler injection

**Figure S3**. Emergency flow diagram for recognising retinal occlusion

**Table S1**. General principles on facial injections with botulinum toxin type A and hyaluronic acid filler products

| Parameter | Steps |
| --- | --- |
| Before treatment | - Patient selection and evaluation |
|  | - - Medical history/medications |
|  | - - Potential contraindications |
|  | - - Previous filler procedures |
|  | - - Other planned procedures |
|  | - Set expectations and explain all potential complications |
|  | - Obtain informed consent (or at time of treatment) |
| Preparation for treatment or injections | - Secure hair from face and cleanse the treatment area of dirt and makeup |
|  | - Use selected pain management methods |
|  | - Apply antiseptic and allow to dry |
|  | - Use the ANTT |
| Posttreatment and follow-up | - Provide specific follow-up instructions and after-hours contact information |
|  | - Schedule follow-up appointments as needed |
|  | - Defer planned surface-active procedures for at most 2−4 weeks after filler treatment and 1 day after BoNTA treatment |

ANTT, Antiseptic Non Touch Technique; BoNTA, botulinum toxin type A.

**Figure S1.** Aseptic Non Touch Technique (ANTT)

**Figure S2**. Recognising the signs and symptoms of vision impairment caused by hyaluronic acid filler injection


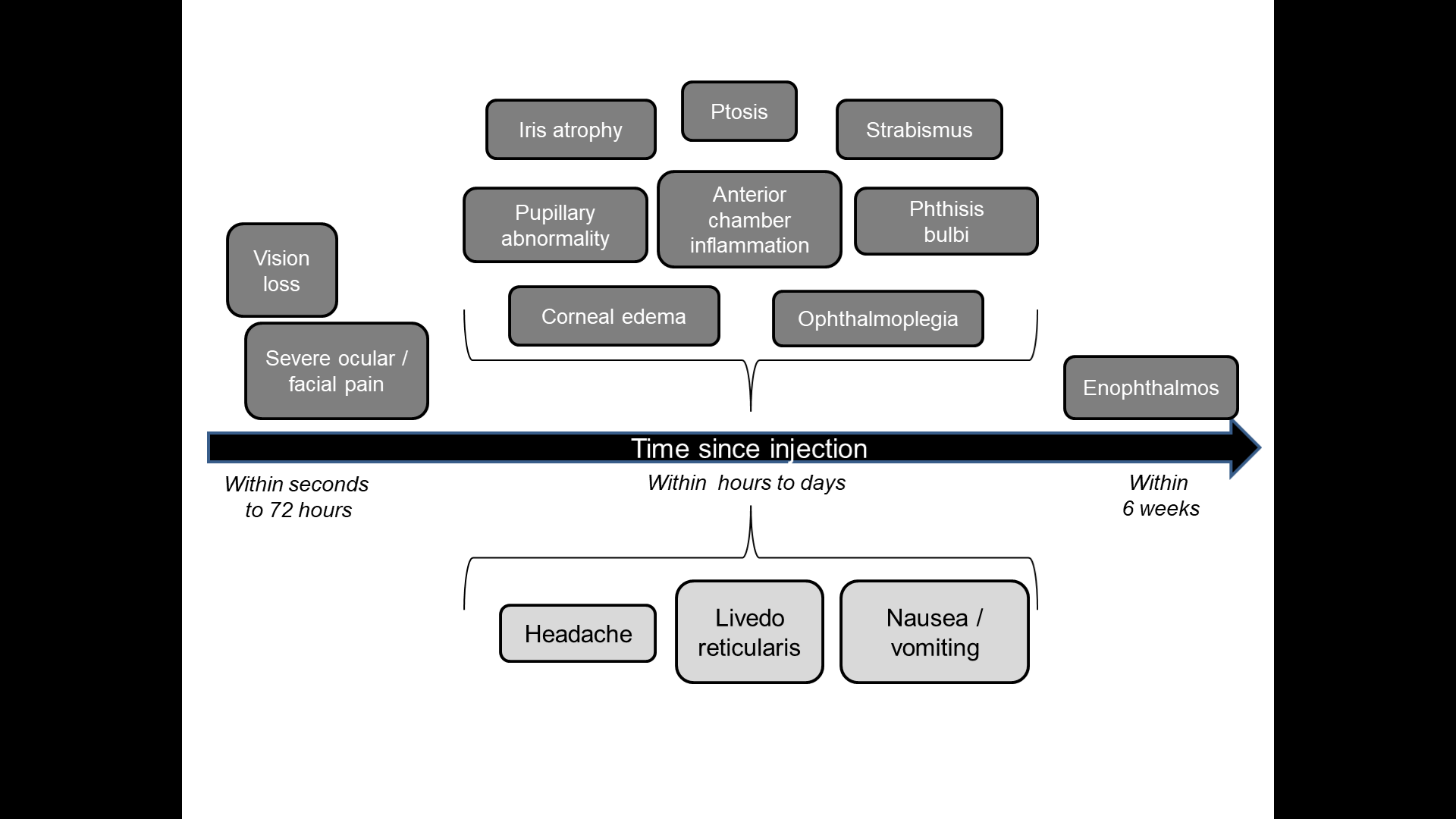


**Figure S3**. Emergency flow diagram for recognising retinal occlusion

*History should include history of migraines; visual defect should be tested in both eyes, separately.

^†^Emergency department (ED) specialists with ophthalmology background OR at an eye hospital preferred.
